# Association between hysterectomy status and stroke risk and cause-specific and all-cause mortality: evidence from the 2005–2018 National Health and Nutrition Examination Survey

**DOI:** 10.3389/fneur.2023.1168832

**Published:** 2023-05-18

**Authors:** Ruihuan Shen, Jia Wang, Yuqing Tian, Rui Wang, Peiyao Guo, Shuhui Shen, Donghao Liu, Tong Zou

**Affiliations:** ^1^Department of Cardiology, Beijing Hospital, National Center of Gerontology, Institute of Geriatric Medicine, Chinese Academy of Medical Sciences & Peking Union Medical College, Beijing, China; ^2^Graduate School of Peking Union Medical College, Beijing, China; ^3^Department of Cardiology, National Center of Gerontology, Institute of Geriatric Medicine, Beijing Hospital, Peking University Fifth School of Clinical Medicine, Beijing, China

**Keywords:** hysterectomy, stroke, women's health issues, NHANES, mortality

## Abstract

**Background:**

Prior research on women who had hysterectomies has shown mixed results on whether or not hysterectomies increased the incidence of stroke and cause-specific or all-cause mortality.

**Methods:**

Using information from the Continuous National Health and Nutrition Examination Survey (NHANES) in the United States, including linked mortality follow-up files available for public access, a multicycle cross-sectional design mortality linkage study was performed.

**Results:**

Conducted during the years 2005–2018, the study sample included 14,214 female participants ranging in age from 20 to 85 years. The relationship between the hysterectomy status and the risk of stroke and cause-specific and all-cause mortality was examined using a series of weighted logistic regressions and Cox proportional hazards regressions, respectively. The presence of a hysterectomy was consistently linked to an elevated risk of stroke using weighted logistic regression models. The hysterectomy status, however, consistently showed no effect on survival by adjusted weighted Cox regression analysis.

**Conclusion:**

Our study found a significant association between hysterectomy and stroke, even after adjusting for other factors that could impact risk, such as the American Heart Association (AHA)'s Life's Simple 7 cardiovascular health score and variables of age, ethnicity, marital status, income, education, and depression severity.

## 1. Introduction

Hysterectomy is the second most prevalent major gynecologic procedure for women in Western countries, following cesarean delivery (1–3). The hysterectomy procedure is a preferred and definite treatment option for many gynecological conditions owing to its low perioperative morbidity and cost-effectiveness. Despite the emergence of minimally invasive treatment alternatives for disorders in recent years, hysterectomy incidence rates in the United States and Western European countries have remained relatively stable (4–9). An estimated 6,00,000 women in the United States undergo this surgery every year (7).

With a 78-year average life expectancy for women in the US, long-term health concerns are crucial. The mean age of the three types of hysterectomies (laparoscopic, abdominal, and vaginal) is below 50 years (10, 11). With increasing life expectancy, hysterectomized women face a long-term impact from surgery (12). Increased awareness of the long-term repercussions of hysterectomy is essential, given that the majority of hysterectomies are performed in perimenopausal women on relative indications (7, 8). Cardiovascular disease (CVD), the leading cause of mortality in women throughout the world, is a case in point (13). The repercussions of hysterectomy should thus be carefully evaluated (14).

Stroke ranks among the top causes of disability and mortality in women and negatively affects their quality of life. High blood pressure, smoking, being overweight, high cholesterol, and diabetes are all major contributors to stroke risk (15). Endogenous sex hormone deficiency is another risk factor for stroke. Women have a decreased risk of stroke in their middle years, whereas menopause is a period when many women develop CVD risk factors, and the risk of stroke in women almost doubles in the decade after menopause (16, 17).

It has been demonstrated that women who undergo hysterectomies, involving those with intact ovaries, have lower levels of endogenous sex hormones than women who do not undergo hysterectomies (18–20). Concern regarding the long-term health effects of hysterectomy is inevitably sparked by these findings.

Due to inconsistent relationships in studies involving hysterectomized women (21–24), it is crucial to validate the relationship between hysterectomy and the risk of stroke and cause-specific and all-cause mortality in a more definitive study population and with a more robust study design. This may provide better evidence for the obstetrician and gynecologist to make clinical decisions and choose the optimal surgical procedures.

With the use of prospectively collected data from the NHANES, this population-based multicycle cross-sectional design and mortality linkage study was conducted nationally with the intention of determining the lifetime risk of stroke after hysterectomy, with or without oophorectomy, after taking into consideration conventional risk factors. In addition, we evaluated the association between hysterectomy and cause-specific and all-cause death, respectively.

## 2. Materials and methods

### 2.1. Database

The National Center for Health Statistics (NCHS) of the Centers for Disease Control and Prevention (CDC) launched numerous cycles of the United States cross-sectional Continuous NHANES from 2005 to 2018 and provided linked mortality follow-up files until 31 December 2019 for public use (25). In addition, the National Center for Health Statistics (NCHS) has connected many demographic surveys to death certificate information from the National Death Index (NDI) (26).

The NHANES used a complex, stratified, multistage, probability cluster design to create a nationally representative survey of the health and nutritional status of the non-institutionalized civilian population in the United States, with detailed information available in the NHANES survey methods and analytic guidelines (27). Additionally, data on the nutritional health and condition of non-institutionalized civilians in the US population were acquired via a series of home interviews, examinations, and laboratory measurements.

After the files were processed to minimize the likelihood of participant identification, the public-use versions of the linked mortality follow-up files provided the mortality data for adult participants, which consisted of mortality follow-up data from the date of survey participation to 31 December 2019 (26).

### 2.2. Study design and population

**Figure 1:**
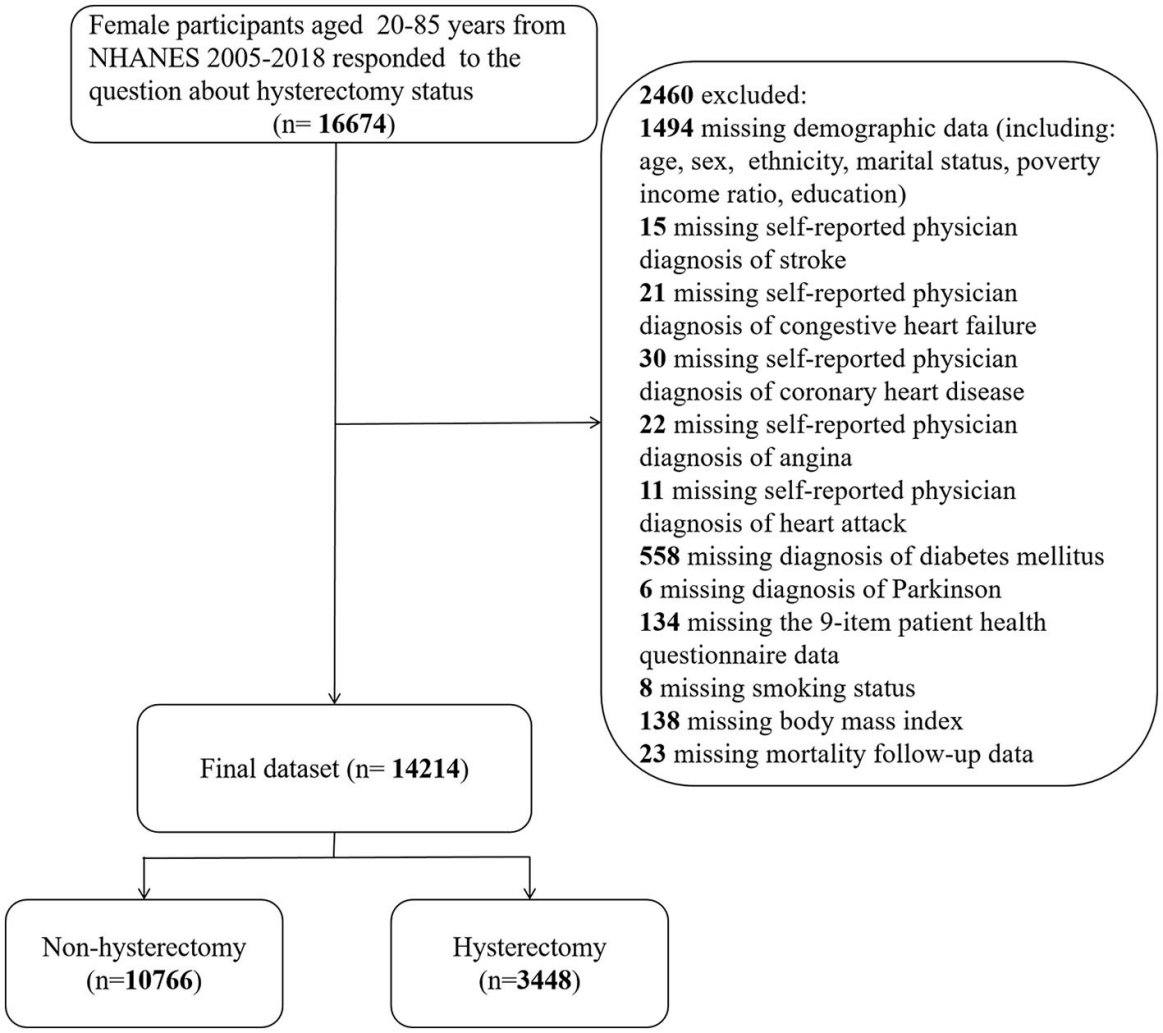
Flowchart of study participants.

Continuous NHANES was used to collect data from 2005 to 2018 in 2-year increments for the initial sample. Only participants with available demographic data who answered the following self-reported questions were included: “Have you ever been told that you had a stroke?” (question MCQ160f of the “Medical Conditions” section) and “Have you ever had a hysterectomy?” (question RHD280 of the “Reproductive Health” section). Responses marked as “missing,” “refused,” or “do not know” were regarded as missing in the original NHANES surveys. Participants who lacked information for any of the study covariates specified below were excluded from the data analysis (Figure 1).

### 2.3. Data collection and weight selection

Data on demographics, physical measures, and comorbidities were recorded. When conducting a household interview, demographic data such as age, ethnicity, marital status, educational level, and certain comorbid conditions were obtained. Trained health technicians and interviewers were arranged to deliver standardized body measurements [e.g., body mass index (BMI)] and questionnaires (e.g., depression severity) to survey participants at the mobile examination facility (MEC). The NHANES 2005–2018 MEC exam data weights were used in all analyses to take stratification and clustering into account because of the complex sample design.

### 2.4. Primary study variables

#### 2.4.1. Assessment of stroke

“Has a doctor or other health professional ever told {you/SP} that {you/s/he}… had a stroke?” was a question on the medical conditions section of the household questionnaires via home interview and those who answered “yes” were deemed to have a history of stroke.

### 2.5. Independent variable

#### 2.5.1. Assessment of hysterectomy status

Participants who responded “yes” to the question “Have you/Has SP had a hysterectomy that is, surgery to remove {your/her} uterus or womb?” on the reproductive health section of the household questionnaires via home interview were deemed as having undergone hysterectomy.

### 2.6. Covariates and confounders

It was necessary to account for a number of possible confounding factors. Age and Life's Simple 7 cardiovascular health score were included in the analysis as continuous variables. The “Life's Simple 7” criteria, devised by the AHA to describe ideal cardiovascular health, included not smoking, regular physical activity, healthy diet, maintaining normal body weight, and controlling cholesterol, blood pressure, and blood glucose levels. The Life's Simple 7 cardiovascular health score varied from 0 to 14 (0 was the worst score and 14 was the optimal score) and was calculated by adding the number of ideal health metrics achieved. In the classification of ethnicity, White, Black, Mexican, and other races were included. The marital status category included married, living with a partner, separated, divorced, widowed, and never married. The educational background was specified as college graduate or above, some college or AA degree, high school graduate, 9–11th grade, or <9th grade. There were three distinct categories of income: low income [poverty income ratio (PIR) <1.3], middle income (PIR = 1.3–3.5), and high income (PIR ≥ 3.5). The categories for smoking status were former smoker, current smoker, and never smoked. BMI was classified as low (i.e., <18.5), normal (i.e., 18.5–25), or overweight (i.e., ≥25). The 9-item patient health questionnaire (PHQ-9) was used to determine the severity of depression: scores of 5, 10, 15, and 20 were used as the thresholds for mild, moderate, moderately severe, and severe depression, respectively.

### 2.7. Comorbid conditions

Information on comorbidities was self-reported by participants. Regarding the question “Have you ever been told by a doctor or health professional that you have …?”, persons who answered “yes” were perceived as having the following comorbidities: coronary heart disease (CHD), congestive heart failure (CHF), heart attack, and angina/angina pectoris.

Hypertension was diagnosed by the following blood pressure/cholesterol questions: BPQ 020: Have you ever been told that you had high blood pressure; BPQ 030: Have you been told that you had high blood pressure 2+ times; BPQ 040a: Are you taking a prescription for hypertension; Are you using an anti-hypertension drug; and Are you judging hypertension on average blood pressure? Average blood pressure was calculated by the following protocol: 1. If only one blood pressure reading was obtained, then that reading was the average. 2. If there was more than one blood pressure reading, then the first reading was always excluded from the average. 3. If only two blood pressure readings were obtained, then the second blood pressure reading was the average. 4. If all the diastolic readings were zero, then the average would be zero.

Chronic obstructive pulmonary disease (COPD) was diagnosed by the following criteria: forced expiratory volume in one second (FEV1)/forced vital capacity (FVC) <0.7 post-bronchodilator; MCQ160p: Has anyone ever told you that you had emphysema; drug use: selective phosphodiesterase-4 inhibitors, mast cell stabilizers, leukotriene modifiers, or inhaled corticosteroids; age above 40; having a smoking history; or having chronic bronchitis.

In addition, Parkinson's disease was diagnosed by taking anti-Parkinson agents, and the diagnostic criteria for diabetes were as follows: a doctor told you that you have diabetes; a glycohemoglobin HbA1c (%) value of >6.5; a random blood glucose concentration (mmol/L) of ≥11.1; a 2-h oral glucose tolerance test (OGTT) blood glucose concentration (mmol/L) of ≥11.1; or the use of diabetes medication or insulin.

### 2.8. Follow-up and outcomes

The follow-up period lasted from the date of the interview through the last follow-up time, 31 December 2019, or the date of death, whichever came first. Records from the NDI provided information on the causes of death for the included participants. The endpoints for this study were as follows: all-cause mortality, which encompassed all known and unknown causes; cardiovascular mortality, which encompassed causes of death related to heart and cerebrovascular disease; and malignant neoplasm mortality.

### 2.9. Statistical analysis

Categorical variables were expressed as weighted proportions and corresponding 95% confidence intervals (CIs). Design-based χ^2^ tests were used to investigate whether categorical variables were associated with hysterectomy status.

The Kolmogorov–Smirnov test of normality was conducted on the continuous variables to determine their distribution (normal or non-normal). Continuous variables with normality were presented as weighted means with associated standard errors (SE), and variables without normality were presented as weighted median with an associated interquartile range (IQR). Furthermore, the two-sample Student's *t-*test was used for normally distributed variables, while the Mann–Whitney *U*-test was used for non-parametric variables.

To determine if the relationship between stroke and hysterectomy status varied across various subgroups of each category of study covariates, an independent stratification analysis was performed. The Wald test was used to calculate the *P*-value for the interaction.

Survival curves were calculated by the weighted Kaplan–Meier method, and the Mantel–Cox log-rank test was adopted to test for differences. The survival probabilities were estimated as the time intervals from the date of the initial interview to the last follow-up time, 31 December 2019, or the date of death.

A series of weighted logistic regression analyses were conducted to assess the association between stroke risk and hysterectomy status in various models after adjusting for potential confounders. Crude and adjusted odds ratios (ORs) and their 95% CIs between stroke risk and hysterectomy status were reported. Similarly, a series of weighted Cox regression analyses were conducted to estimate the association between hysterectomy status and the probabilities of cause-specific and all-cause death after controlling for possible confounding factors in various models. The correlation between the hysterectomy status and outcomes was provided as a crude and adjusted hazard ratio (HR) and its 95% CIs. The multivariable model included the following confounders based on previous studies: age (continuous), ethnicity (White, Black, Mexican, or other), marital status (married, living with a partner, separated, divorced, widowed, and never married), poverty income ratio [classified as low income (<1.3), middle income (1.3–3.5), and high income (≥3.5)], educational level (divided into <9th grade, 9–11th grade, high school graduate, some college or AA degree, and college graduate or above), depression severity (none, mild, moderate, moderately severe, and severe), and AHA's Life's Simple 7 cardiovascular health score. The first model is the unadjusted model, and another adjustment model with robust adjustment for covariates is thought to be the potential confounder of the association of hysterectomy status with stroke risk in weighted logistic regression analyses, and with all-cause, cardiovascular, and malignant neoplasm mortality in weighted Cox regression analyses.

For statistical analysis, R (version 4.1.2; https://www.R-project.org) was used. The complexity of the sampling design was taken into account in each analysis by specifying primary sampling units (PSUs), strata, and weights using the R package “survey” (version 4.1-1). We used MEC exam weights for all sample estimations (28–30). A result with a two-sided *P*-value of <0.05 was considered statistically significant when testing the hypotheses of the study.

## 3. Results

### 3.1. Participant characteristics

According to the inclusion and exclusion criteria, the unweighted sample for the final analysis consisted of 14,214 female participants aged 20–85 years from 2005 to 2018, representing 87.69 million non-institutionalized United States residents. A total of 3,448 participants (a weighted proportion of 23.37%) underwent hysterectomy, while 576 participants (a weighted proportion of 3.29%) had a stroke, corresponding to 20.49 million and 2.88 million adults in the general population, respectively.

Table 1 describes the sociodemographic and clinical characteristics of the weighted population. Notably, compared to participants in the non-hysterectomy group, hysterectomy was more prevalent among older white female participants with a lower Life's Simple 7 cardiovascular health score and educational background; a higher PHQ-9 score; former smoking status; overweight status; middle income; married, divorced, or widowed marital status; and greater risk of the following comorbidities: stroke, hypertension, CHF, CHD, angina, heart attack, COPD, diabetes, and Parkinson's disease.

**Table 1:** Baseline characteristics of study participants^a^.

**Characteristic**	**Participants**			**P -value**
	**Total (*****n*** = **14,214)**	**Non-hysterectomy (*****n*** = **10,766)**	**Hysterectomy (*****n*** = **3,448)**	
Age	49.18 ± 0.28	45.46 ± 0.28	61.38 ± 0.34	<0.0001
Life's simple 7	8.28 ± 0.04	8.60 ± 0.04	7.25 ± 0.05	<0.0001
Ethnicity (%)				<0.0001
White	69.65 (64.22, 75.09)	67.67 (64.93, 70.41)	76.16 (73.53, 78.79)	
Black	11.27 (10.03, 12.51)	11.15 (9.66, 12.64)	11.66 (9.89, 13.43)	
Mexican	7.03 (5.88, 8.18)	7.96 (6.59, 9.32)	3.99 (2.96, 5.03)	
Other	12.05 (11.13, 12.96)	13.22 (11.97, 14.48)	8.18 (6.96, 9.41)	
Marital status (%)				<0.0001
Married	52.39 (49.10, 55.68)	51.10 (49.55, 52.65)	56.62 (54.08, 59.15)	
Living with partner	7.31 (6.64, 7.97)	8.62 (7.90, 9.34)	3.00 (2.15, 3.85)	
Separated	2.61 (2.31, 2.91)	2.72 (2.34, 3.09)	2.25 (1.71, 2.79)	
Divorced	12.81 (11.88, 13.74)	11.93 (11.12, 12.74)	15.69 (14.02, 17.37)	
Widowed	9.18 (8.47, 9.88)	6.52 (6.00, 7.05)	17.88 (16.14, 19.62)	
Never married	15.71 (14.52, 16.89)	19.11 (17.73, 20.49)	4.55 (3.66, 5.45)	
Educational level (%)				<0.0001
College graduate or above	30.04 (27.43, 32.65)	32.96 (30.88, 35.04)	20.48 (18.11, 22.85)	
Some college or AA Degree	33.59 (31.71, 35.47)	33.02 (31.65, 34.39)	35.46 (32.95, 37.96)	
High school graduate	22.46 (21.02, 23.90)	20.94 (19.63, 22.24)	27.47 (25.39, 29.55)	
9–11th grade	9.62 (8.73, 10.51)	9.01 (8.20, 9.82)	11.62 (10.21, 13.02)	
<9th grade	4.28 (3.80, 4.77)	4.07 (3.55, 4.60)	4.98 (4.14, 5.81)	
Poverty income ratio (%)^b^				<0.001
High income	41.53 (38.33, 44.72)	42.01 (39.73, 44.30)	39.93 (37.31, 42.54)	
Middle income	36.26 (34.17, 38.35)	35.08 (33.58, 36.59)	40.12 (38.05, 42.19)	
Low income	22.21 (20.86, 23.57)	22.90 (21.35, 24.46)	19.95 (18.22, 21.68)	
Smoking status (%)^c^				<0.0001
Never	60.81 (58.09, 63.52)	62.56 (61.01, 64.10)	55.09 (52.61, 57.56)	
Former	21.31 (19.57, 23.06)	19.52 (18.23, 20.82)	27.19 (24.74, 29.63)	
Current	17.88 (16.51, 19.24)	17.92 (16.79, 19.05)	17.73 (15.75, 19.70)	
Body mass index (%)^d^				<0.0001
Normal	30.62 (28.62, 32.62)	32.98 (31.52, 34.44)	22.89 (20.90, 24.88)	
Overweight	67.38 (64.17, 70.59)	64.76 (63.26, 66.27)	75.97 (73.96, 77.97)	
Low	1.99 (1.70, 2.29)	2.25 (1.86, 2.65)	1.15 (0.74, 1.55)	
Depression severity (%)^e^				<0.0001
None	72.58 (69.03, 76.14)	74.49 (73.36, 75.62)	66.33 (64.16, 68.50)	
Mild	17.60 (16.53, 18.66)	16.60 (15.75, 17.46)	20.85 (19.01, 22.68)	
Moderate	6.19 (5.62, 6.77)	5.70 (5.17, 6.23)	7.81 (6.68, 8.93)	
Moderately severe	2.65 (2.26, 3.04)	2.35 (2.00, 2.70)	3.65 (2.70, 4.59)	
Severe	0.97 (0.79, 1.16)	0.86 (0.64, 1.07)	1.36 (0.93, 1.79)	
Comorbidity stroke (%)	3.29 (2.92, 3.66)	2.07 (1.77, 2.36)	7.30 (6.27, 8.33)	<0.0001
Comorbidity hypertension (%)	39.02 (36.93, 41.12)	32.06 (30.76, 33.36)	61.85 (59.92, 63.79)	<0.0001
Comorbidity CHF (%)	2.19 (1.92, 2.47)	1.41 (1.17, 1.66)	4.75 (3.87, 5.62)	<0.0001
Comorbidity CHD (%)	2.30 (1.92, 2.68)	1.49 (1.18, 1.80)	4.96 (4.05, 5.86)	<0.0001
Comorbidity angina (%)	1.93 (1.64, 2.22)	1.27 (1.02, 1.51)	4.10 (3.17, 5.04)	<0.0001
Comorbidity heart attack (%)	2.45 (2.12, 2.77)	1.61 (1.35, 1.86)	5.20 (4.31, 6.10)	<0.0001
Comorbidity COPD (%)	4.50 (3.93, 5.07)	3.28 (2.76, 3.81)	8.47 (7.12, 9.83)	<0.0001
Comorbidity diabetes (%)				<0.0001
No	81.31 (77.41, 85.21)	83.94 (83.00, 84.87)	72.69 (70.97, 74.41)	
Diabetes	13.70 (12.74, 14.67)	11.44 (10.64, 12.23)	21.13 (19.74, 22.51)	
IGT	4.99 (4.48, 5.50)	4.63 (4.16, 5.10)	6.18 (5.14, 7.22)	
Comorbidity PD (%)	1.23 (0.98, 1.47)	0.80 (0.58, 1.01)	2.63 (1.93, 3.33)	<0.0001

CHF, congestive heart failure; CHD, arteriosclerotic cardiovascular disease; COPD, chronic obstructive pulmonary disease; IGT, impaired glucose tolerance; PD, Parkinson's disease; PHQ-9, 9-item patient health questionnaire.

a Two-sided P-values show results of univariate comparisons between hysterectomized female participants and participants who were not hysterectomized. The two-sample Student's t-test was used for normally distributed variables, while Mann–Whitney U-test was used for non-parametric variables. Design-based χ^2^ tests were employed to assess the associations of categorical variables with hysterectomy status.

b Categorized into the following three levels based on the poverty income ratio: low income (<1.3), medium income (1.3–3.5), and high income (≥3.5).

c Categorized into the following three levels: never, smoked <100 cigarettes in life; former, smoked more than 100 cigarettes in life and smoke not at all; and current, smoked more than 100 cigarettes in life and smoke some days or every day.

d Divided into four categories: low (<18.5 kg/m^2^), normal (18.5–25 kg/m^2^), and overweight (≥25 kg/m^2^).

e Cut-points of 5, 10, 15, and 20 of PHQ-9 score represent the thresholds for mild, moderate, moderately severe, and severe depression, respectively.

### 3.2. Subgroup analyses

**Figure 2:**
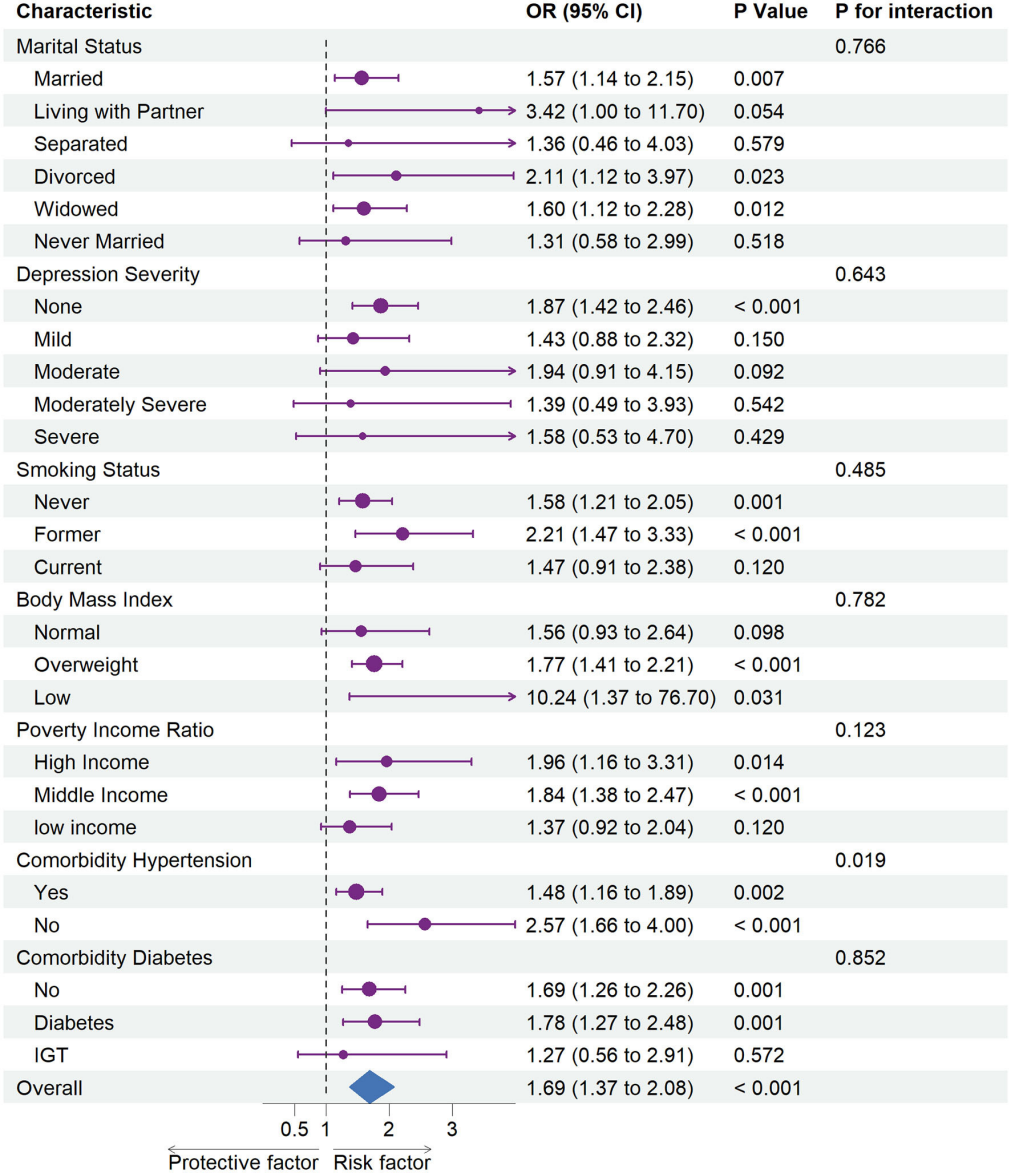
Association between hysterectomy and stroke in subgroups. OR, odds ratio; CI, confidence interval. Each stratification was adjusted for age (continuous), ethnicity (White, Black, Mexican, or other), marital status (married, living with partner, separated, divorced, widowed, or never married), poverty income ratio [classified as low income (<1.3), middle income (1.3–3.5), and high income (≥3.5)], educational level (divided into <9th grade, 9–11th grade, high school graduate, some college or AA degree, and college graduate or above), and depression severity (none, mild, moderate, moderately severe, or severe), and the American Heart Association's Life's Simple 7 cardiovascular health score (continuous), except the stratification factor itself. Squares indicate ORs, with horizontal lines indicating 95% CIs. The center of the diamond indicates the overall OR between stroke risk and hysterectomy status in the overall population, with the outer points of the diamonds indicating 95% CI.

Figure 2 summarizes the results of the subgroup analysis using multivariable-adjusted weighted logistic regressions.

A subgroup analysis demonstrated increased risks of stroke related to hysterectomy status among all participants (OR = 1.69, 95% CI = 1.37–2.08). In particular, an elevated risk of stroke was associated with hysterectomy status among non-depressed (OR = 1.87, 95% CI = 1.42–2.46) participants whose marital status was married (OR = 1.57, 95% CI = 1.14–2.15), divorced (OR = 2.11, 95% CI = 1.12–3.97), or widowed (OR = 1.60, 95% CI = 1.12–2.28) and who never smoked (OR = 1.58, 95% CI = 1.21–2.05) or were former smokers (OR = 2.21, 95% CI = 1.47–3.33). In addition, we identified an elevated risk of stroke associated with hysterectomy status among those with high (OR = 1.77, 95% CI = 1.41–2.21) or low (OR = 10.24, 95% CI = 1.37–76.70) BMI, high (OR = 1.96, 95% CI = 1.16–3.31) or middle (OR = 1.84, 95% CI = 1.38–2.47) income and comorbid diabetes (OR = 1.78, 95% CI = 1.27–2.48) or not (OR = 1.69, 95% CI = 1.26–2.26).

A statistically significant interaction was identified in subgroup analyses between hysterectomy status and hypertension (*P* for interaction = 0.019) in relation to an elevated risk of stroke.

### 3.3. Association between hysterectomy status and stroke risk

The results of weighted logistic regression analyses of hysterectomy status in relation to the risk of stroke are displayed in Table 2. There were significant associations between hysterectomy status and increased risk of stroke in Model 1 (unadjusted model), Model 2 (adjusted for age (continuous), ethnicity (White, Black, Mexican, or other), marital status (married, living with a partner, separated, divorced, widowed, or never married), poverty income ratio [classified as low income (<1.3), middle income (1.3–3.5), and high income (≥3.5)], educational level (divided into <9th grade, 9–11th grade, high school graduate, some college or AA degree, or college graduate or above), and depression severity (none, mild, moderate, moderately severe, or severe), and Model 3 [further adjusted for the American Heart Association's Life's Simple 7 (continuous)]. For example, the result in Model 3 showed that for those who underwent hysterectomy, the risk of having a stroke increased by 69% (OR = 1.69, 95% CI = 1.37–2.08) compared with women with an intact uterus. We also carried out a stratification analysis by hypertension status in women who underwent a hysterectomy. Hysterectomy not only increased the risk of developing stroke in women with hypertension but also increased the risk of developing stroke in women without hypertension.

**Table 2:** Crude and adjusted association between hysterectomy and stroke: overall and stratified to comorbidity hypertension.

**Model**	**Overall**	**Non-hypertension**	**Hypertension**	***P* for interaction**
	**OR (95% CI)**	**OR (95% CI)**	**OR (95% CI)**	**Hysterectomy and hypertension**
Model 1	3.73 (3.03, 4.60)	5.31 (3.51, 8.05)	2.03 (1.58, 2.62)	
*P*-values	<0.001	<0.001	<0.001	<0.001
Model 2	1.71 (1.39, 2.10)	2.59 (1.67, 4.01)	1.47 (1.15, 1.88)	
*P*-values	<0.001	<0.001	0.003	0.013
Model 3	1.69 (1.37, 2.08)	2.57 (1.66, 4.00)	1.48 (1.16, 1.89)	
*P*-values	<0.001	<0.001	0.002	0.019

Model 1: Unadjusted model.

Model 2: Adjusted for age (continuous), ethnicity (White, Black, Mexican, or other), marital status (married, living with partner, separated, divorced, widowed, or never married), poverty income ratio [classified as low income (<1.3), middle income (1.3–3.5), and high income (≥3.5)], educational level (divided into <9th grade, 9–11th grade, high school graduate, some college or AA degree, and college graduate or above), and depression severity (none, mild, moderate, moderately severe, or severe).

Model 3: Further adjusted for the American Heart Association's Life's Simple 7 cardiovascular health score (continuous).

OR, odds ratio.

### 3.4. Survival analysis

The leading causes of death for those with and without a hysterectomy are listed in Table 3. Among them, the prevalence of all-cause, cardiovascular, or malignant neoplasm mortality was 5.09, 1.40, and 1.27% for non-hysterectomized women, respectively; the prevalence of all-cause, cardiovascular, or malignant neoplasm mortality was 13.69, 3.98, and 3.01% for hysterectomized women, respectively.

**Table 3:** Weighted prevalence of leading causes of death in different hysterectomy status.

**Cause of death**	**Non-hysterectomy**	**Hysterectomy**
Diseases of heart (%)	1.16	3.24
Cerebrovascular diseases (%)	0.24	0.74
Influenza and pneumonia (%)	0.11	0.26
Chronic lower respiratory diseases (%)	0.33	0.98
Nephritis, nephrotic syndrome and nephrosis (%)	0.06	0.40
Diabetes mellitus (%)	0.17	0.56
Malignant neoplasms (%)	1.27	3.01
Alzheimer's disease (%)	0.11	0.64
Accidents (unintentional injuries) (%)	0.17	0.12
All other causes (residual) (%)	1.47	3.74
All-cause (%)	5.09	13.69

**Figure 3:**
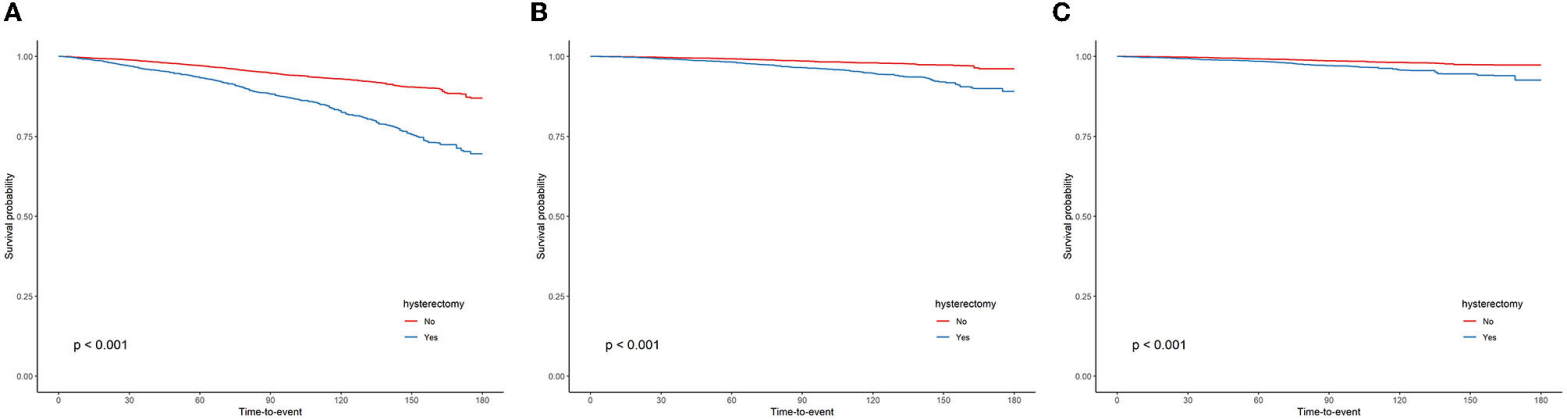
Kaplan–Meier curves were depicted to show the association between the hysterectomy and all-cause **(A)**, cardiovascular **(B)**, and malignant neoplasms **(C)** mortality, with follow-up in months.

The Kaplan–Meier curves for all-cause, cardiovascular, and malignant neoplasm mortality are presented in Figures 3A–C, respectively. The median follow-up time from the date of interview to the last follow-up time, 31 December 2019, or the date of death was 85 months (ranging from 1 to 180 months). The upper survival curves for non-hysterectomized women were all above the lower curve for hysterectomized women across the entire 180 months of follow-up (all log-rank *P* < 0.001), visually indicating that the survival probability of female participants with an intact uterus was greater than that of female participants who underwent hysterectomy and suggesting a survival benefit.

The weighted Cox regression analysis results, which are shown in Table 4, estimated the association between hysterectomy status and the hazard of all-cause, cardiovascular, or malignant neoplasm mortality. Unadjusted weighted Cox regressions indicated that having a hysterectomy was associated with an increased risk of all-cause (HR = 2.56, 95% CI = 2.22–2.96), cardiovascular (HR = 2.68, 95% CI = 2.18–3.28), or malignant neoplasm mortality (HR = 2.28, 95% CI = 1.73–3.02). However, a series of multivariable-adjusted weighted Cox regressions consistently revealed that hysterectomy may not contribute much to the risks of all-cause (HR = 1.01, 95% CI = 0.89–1.14, fully adjusted model), cardiovascular (HR = 0.99, 95% CI = 0.83–1.19, fully adjusted model), or malignant neoplasm mortality (HR = 1.01, 95% CI = 0.75–1.36, fully adjusted model).

**Table 4:** Crude and adjusted association between hysterectomy status and cause-specific and all-cause mortality.

**Model**	**All-cause mortality**	**CVD mortality**	**Malignant neoplasms mortality**
	**HR (95% CI)**	**HR (95% CI)**	**HR (95% CI)**
Model 1	2.56 (2.22, 2.96)	2.68 (2.18, 3.28)	2.28 (1.73, 3.02)
*P*-values	<0.001	<0.001	<0.001
Model 2	1.06 (0.93, 1.21)	1.02 (0.84, 1.24)	1.04 (0.77, 1.42)
*P*-values	0.394	0.825	0.785
Model 3	1.02 (0.89, 1.16)	1.00 (0.83, 1.20)	1.02 (0.75, 1.38)
*P*-values	0.799	0.994	0.904
Model 4	1.01 (0.89, 1.14)	0.99 (0.83, 1.19)	1.01 (0.75, 1.36)
*P*-values	0.910	0.915	0.963

Model 1: Unadjusted model.

Model 2: Adjusted for age (continuous).

Model 3: Adjusted for age (continuous), ethnicity (White, Black, Mexican, or other), marital status (married, living with partner, separated, divorced, widowed, or never married), poverty income ratio [classified as low income (i.e., <1.3), middle income (i.e., 1.3–3.5), and high income (i.e., ≥3.5)], educational level (divided into <9th grade, 9–11th grade, high school graduate, some college or AA degree, or college graduate or above), and depression severity (none, mild, moderate, moderately severe, or severe).

Model 4: Further adjusted for the American Heart Association's Life's Simple 7 cardiovascular health score (continuous).

HR, hazard ratio; CVD, cardiovascular disease.

## 4. Discussion

It was imperative to note that although hysterectomy can be an appropriate therapeutic option for some women, the effects of hysterectomy may not be reversible over the course of their lives. Any long-term impact of hysterectomy should therefore be considered (31).

Our study found that hysterectomy was more prevalent among older white female participants with a lower Life's Simple 7 cardiovascular health score, lower educational background, higher PHQ-9 score, former smokers, overweight status, whose earnings landed in the middle, and whose marital status was married, divorced, or widowed. The results indicated that marital status, education, and income level are important factors for those at risk. Age, race, BMI, depression severity, smoking status, and Life's Simple 7 cardiovascular health score may also play a role in women who underwent hysterectomy. Thus, these confounders were gradually adjusted in a series of Cox proportional hazards regressions and weighted logistic regressions to confirm the association among hysterectomy status with stroke risk, cause-specific mortality, and all-cause mortality.

Therefore, we may be able to demonstrate an increased risk of stroke in women having a hysterectomy, which could have global and profound implications for women's health. Women's healthcare providers can take advantage of these findings to improve screenings for cardiovascular health.

The results of our study were consistent with those of the following studies regarding hysterectomy status and stroke. Studies have previously been performed to understand the association between hysterectomy status and CVD risks (32, 33). Recently, several cohort studies have investigated the risk of stroke and hysterectomy status. An association between hysterectomy and stroke among women under 50 years old at study entry was found in a nationwide study in Sweden (HR = 2.22, 95% CI = 1.001–4.83) (22). A cohort study conducted in Taiwan found that stroke risk was significantly elevated among women who had a hysterectomy before 45 years of age (HR = 2.29, 95% CI = 1.52–3.44) after controlling for baseline cardiovascular risk factors (34).

As of now, it is not known what pathophysiological mechanisms linked hysterectomy by itself to an increased stroke risk among women with intact uteruses. It is likely that hormone-related effects on the vascular bed and ovarian failure are responsible for the association between hysterectomy status and stroke. There have been several longitudinal studies showing that hysterectomy caused early menopause and premature ovarian failure (35). In comparison with premenopausal women without hysterectomy, women with ovarian preservation at the time of hysterectomy reported higher levels of follicle-stimulating hormone and lower levels of ovarian sex steroids (18–20). There was evidence to support the hypothesis that the disruption of the ovarian blood flow from the uterus led to accelerated ovarian follicular depletion and diminished ovarian reserve (36). As a consequence of hormone-related effects on the vascular bed, atherosclerosis may be exacerbated. Thus, having a hysterectomy before 45–50 years of age was associated with an increased risk of stroke in the two studies mentioned above, suggesting that accelerated estrogen deficiency was associated with stroke.

A large observational study of the Women's Health Initiative, in contrast to those above, found that hysterectomy did not have a significant effect on CVD after adjustment for demographics and risk factors, which suggested that higher cardiovascular risk associated with hysterectomy may be attributed to the more adverse initial risk profile of hysterectomized women rather than the operation itself (21). We found that our results were inconsistent with those of Howard et al. (21), which may be attributed to the different populations and incident CVD outcomes used in our study. Howard et al. confined their study to post-menopausal women, while we incorporate all women into our study; furthermore, the incident CVD outcome in their study includes coronary death, myocardial infarction, stroke, and coronary revascularization procedures, whereas our study focuses exclusively on stroke. Some studies have reported contradictory results. In a systematic review and meta-analysis, hysterectomies were found to provide some protection against stroke (relative risk (RR) = 0.88, 95% CI = 0.85–0.90). The study, however, did not provide any information about the reason why this occurred (37). It is noteworthy that two studies that did not conclude that women with hysterectomy had an increased risk of stroke were characterized by small sample sizes or evaluations using risk factors for CVD that did not include stroke. Thus, these two studies were weak in determining the relationship between hysterectomy and stroke (24, 38).

It is interesting to mention that various studies have shown that hypertension was one of the most important risk factors for stroke (39). Remarkably, other than that, we also found there were hysterectomy status and hypertension interaction effects on the stroke. Nevertheless, a series of weighted logistic regressions consistently revealed that whether it was in all the participants, participants with hypertension, or participants without hypertension, there was an increased risk of stroke in women having a hysterectomy, which suggested that the association between the risk of stroke and hysterectomy status was likely to be independent of hypertension.

The fact that the age-adjusted and multivariable-adjusted HRs for all-cause, cardiovascular, or malignant neoplasms mortality were dramatically lower than the crude HRs was a strong indicator of the reason why the survival probability of hysterectomized women was significantly lower than non-hysterectomized women in Kaplan–Meier analyses was that hysterectomized women are older than non-hysterectomized women (61.38 ± 0.34 vs. 45.46 ± 0.28, *P* < 0.0001) in our study. For this reason, hysterectomy had not increased the risks of all-cause, cardiovascular, or malignant neoplasms mortality.

There are some limitations to the present study that deserve attention. First, the application of the competitive risk model in the survival analyses cannot be performed due to the complex, stratified multistage, probability cluster design of the NHANES survey. The second point to consider is whether there is any possibility that some residual and unmeasured confounders exist, which might bias the findings of our study, even though we have controlled most of the cardiovascular risk factors using weighted logistic regressions and Cox proportional hazards regressions. Third, hysterectomy and stroke status were all obtained from self-report, which may result in recall bias or interviewer bias. Fourth, there is a large proportion of missing values in combined oral contraceptive and hormone replacement therapy used in NHANES; this will lead to the loss of a large sample size of our study, which will result in insufficient statistical power of this study; thus, by comprehensive consideration, hormone replacement therapy use had not been accounted for in the analysis. Last but not least, this is a cross-sectional study, so whether hysterectomy was done before the stroke occurred cannot be ascertained; as a consequence, causality should not be claimed on the basis of these findings.

## 5. Conclusion

In conclusion, our study confirmed that hysterectomy status was strongly linked to living with stroke, even after adjusting for other factors that could impact risk, such as the AHA's Life's Simple 7 cardiovascular health score and variables of age, ethnicity, marital status, income, education, and depression severity. Thus, in the United States, increased attention should be placed on preventing stroke in women who have undergone hysterectomy.

## Data availability statement

The original contributions presented in the study are included in the article/supplementary material, further inquiries can be directed to the corresponding author.

## Ethics statement

Ethical review and approval was not required for the study on human participants in accordance with the local legislation and institutional requirements. Written informed consent from the patients/participants or patients/participants' legal guardian/next of kin was not required to participate in this study in accordance with the national legislation and the institutional requirements.

## Author contributions

RS: full access to all of the data in the study and takes responsibility for the integrity of the data and the accuracy of the data analysis, and drafting of the manuscript. RS and JW: concept and design. RS, TZ, JW, PG, and SS: critical revision of the manuscript for important intellectual content. RS and SS: statistical analysis. TZ: obtained funding and supervision. TZ, DL, and PG: administrative, technical, and material support. All authors acquisition, analysis, and interpretation of data. All authors have read and approved your manuscript.
